# Early and long-term effects of prophylactic and post-excision human papillomavirus vaccination on recurrent high-grade cervical intraepithelial neoplasia relative to margin status: a retrospective cohort study in the Czech Republic

**DOI:** 10.1016/j.lanepe.2025.101337

**Published:** 2025-06-03

**Authors:** Marek Petráš, Danuše Lomozová, Vladimír Dvořák, Vladimír Dvořák, Jana Malinová, Markéta Trnková, Ivan Fišer, Pavel Dlouhý, Jozef Rosina, Ivana Králová Lesná

**Affiliations:** a Department of Epidemiology and Biostatistics, Third Faculty of Medicine, Charles University, Prague 100 00, Czech Republic; b Centre of Ambulatory Gynaecology and Primary Care, Brno 602 00, Czech Republic; c Department of Obstetrics and Gynaecology, Palacký University Olomouc, Faculty of Medicine and Dentistry, University Hospital Olomouc, Olomouc 775 15, Czech Republic; d Královské Vinohrady University Hospital, Prague 100 00, Czech Republic; e Unilabs Pathology k.s., Prague 160 00, Czech Republic; f Department of Hygiene, Third Faculty of Medicine, Charles University, Prague 100 00, Czech Republic; g Department of Medical Biophysics and Informatics, Third Faculty of Medicine, Charles University, Prague 100 00, Czech Republic; h Department of Health Care and Population Protection, Faculty of Biomedical Engineering, Czech Technical University in Prague, Kladno 272 01, Czech Republic; i Laboratory for Atherosclerosis Research, Centre for Experimental Medicine, Institute for Clinical and Experimental Medicine, Prague 140 21, Czech Republic; j Department of Anaesthesia and Intensive Medicine, First Faculty of Medicine, Charles University and University Military Hospital, Prague 100 00, Czech Republic

**Keywords:** Adjuvant intervention, Recurrence, Human papillomavirus, Vaccination, Conisation, Cervical intraepithelial neoplasia

## Abstract

**Background:**

The effect of human papillomavirus (HPV) vaccination on cervical intraepithelial neoplasia grade 2 or worse (CIN2+) recurrence with respect to cone margin positivity is unknown. Most studies assessed this effect beyond two months post-conisation. We aimed to determine both the duration and early onset of effect in women who had been prophylactically vaccinated or vaccinated after conisation, considering cone margin status.

**Methods:**

This cohort study used data from one of the central laboratories in the Czech Republic, covering approximately 33% of women undergoing national cervical cancer screening. It included women treated for CIN2+ between 2010 and 2024 who had received either prophylactic HPV vaccination (available through the national immunisation program since 2011) or post-conisation vaccination (recommended by the Czech Gynaecological and Obstetrical Society since 2008). The vaccination effect was estimated using the incidence rate ratio (IRR) from a Poisson regression model, calculated as 100 × (1–IRR).

**Findings:**

Of the 10,054 women enrolled, 919 were vaccinated after conisation, 502 prophylactically, and 169 had undetermined timing of vaccination. Throughout the follow-up period, CIN2+ recurrence was observed in 513 unvaccinated women, with a rate of 14·61 per 1000 person-years (py), in 14 prophylactically vaccinated women, with a rate of 5·84 (54% reduction; 95% confidence interval [CI]: 22–73%), and in 15 women vaccinated post-excision, with a rate of 3·37 (74% reduction; 95% CI: 57–85%). The high recurrence rate of 58·59 per 1000 py within six months of conisation was reduced by 80% (95% CI: 19–95%) with prophylactic vaccination and by 89% (95% CI: 57–97%) with incomplete post-excision vaccination. Among a total of 1771 women with a positive cone margin, recurrence was identified in 272 of 1568 unvaccinated women, corresponding to a recurrence rate of 51·62 per 1000 py. A reduction was observed in 84 prophylactically vaccinated and in 119 women vaccinated post-excision, with only 6 recurrence cases documented in each group. This corresponded to recurrence rates of 14·94 (62% reduction; 95% CI: 14–83%) and 9·78 per 1000 py (79% reduction; 95% CI: 52–90%), respectively.

**Interpretation:**

Regardless of timing, HPV vaccination has a beneficial long-term effect in lowering the risk of CIN2+ recurrence. Despite the greater reduction in relapse achieved by post-excision vaccination, the difference compared with the prophylactic one was not statistically significant. The most pronounced benefit was observed within the first six months post-conisation, particularly in women with a positive cone margin.

**Funding:**

Cooperatio 31 fund, Health Sciences, 10.13039/100007397Charles University, Prague, Czech Republic.

**Research in context**

**Evidence before this study**

In 2023, we published a quantitative synthesis of studies on the effect of HPV vaccination in women who had undergone excision. A computerized literature search was conducted to identify publications issued between 1 January 2001 and 25 May 2023 containing the keywords “immunisation”, “HPV”, and “effectiveness”, or synonyms thereof. The search utilized the MEDLINE, EMBASE, International Pharmaceutical Abstracts, Derwent Drug File, and ProQuest Science and Technology databases via the PubMed, STNext, Cochrane platforms, and MedRxiv.

The pooled vaccine effectiveness from 21 estimates across 20 eligible studies was 69·5% (95% CI: 54·7–79·5%), regardless of vaccination timing. The greatest effect, 78·1% (95% CI: 68·7–84·7%), was seen in women who completed vaccination after excision, with a prediction interval of 67·1–85·4%. In women vaccinated before excision, CIN2+ recurrence were reduced by 49·8% (95% CI: −45·5 to 82·7%). Our recently published 2024 study, which showed a 90% reduction in CIN2+ recurrence (95% CI: 12–99%) in 9vHPV-vaccinated women post-conisation, was absent from this synthesis.

To date, no studies have evaluated the early effect of vaccination immediately after conisation or its impact in women with positive or negative cone margins. Moreover, follow-up duration in most studies was limited to 4 years.

**Added value of this study**

The present study demonstrated the benefit of HPV vaccination completed both after and before conisation, with a stronger effect observed with post-conisation vaccination, aligning with the outcomes of our meta-analysis. For the first time, vaccination was shown to significantly reduce the high recurrence risk in women with a positive cone margin, lowering it to a level comparable with that of unvaccinated women with a negative margin. A novel finding was the early onset of the HPV vaccination effect, observed within the first six months of conisation, even in women who had not yet completed vaccination. Data spanning 15 years demonstrated the persistence of this effect for six or more years.

**Implications of all the available evidence**

Our findings underscore the importance of HPV vaccination in women undergoing cervical conisation, particularly those with positive cone margins who are at high risk of recurrence. Vaccination significantly reduces the likelihood of recurrence, which are most common within the first six months of surgical excision. These new findings represent a breakthrough in reducing recurrence risks for the most vulnerable group of women during the high-risk period. This may contribute to increased adherence to vaccination following cervical conisation. Additionally, our results highlight the added benefit of HPV vaccination in adolescent and young women, further supporting its promotion at a younger age.

## Introduction

Since its introduction in 2006, vaccination against human papillomavirus (HPV)-related diseases has been incorporated into immunisation programs worldwide. Initially recommended for women aged 9–26 years, its use has expanded to include older women (since 2010), boys (since 2009), and young men (since 2013) to prevent cervical, vaginal, vulvar, anal, penile, and oropharyngeal cancers. The greatest benefits are seen when vaccination occurs before sexual debut.^1^ A successful strategy of HPV vaccination relies on vaccine availability and, since 2006, three bivalent (2vHPV), two quadrivalent (4vHPV), and one nonavalent (9vHPV) vaccines have been available globally. All target high-risk HPV genotypes 16 and 18, with 4vHPV and 9vHPV vaccines covering also low-risk genotypes 6 and 11, and 9vHPV including five additional high-risk genotypes: 31, 33, 45, 52, and 58.^2^

In the Czech Republic, prophylactic HPV vaccination was incorporated into the national immunisation program in 2011, initially for 13-year-old girls with a three-dose regimen administered over six months. It was later expanded to include both boys and girls aged 11–14, following a two-dose regimen given over 6–12 months. Since 2008, the Czech Gynaecological and Obstetrical Society has recommended post-conisation vaccination to reduce the risk of disease from other HPV genotypes, and in 2014, this was further specified to address dysplasia recurrence.^3^

The risk of HPV-related disease recurrence increases over time after treatment.^4^ Over the past 15 years, research has emphasized the role of HPV vaccination, particularly in reducing the recurrence of cervical intraepithelial neoplasia grade 2 or higher, and cervical carcinoma (CIN2+). According to the WHO classification, CIN2+ corresponds to high-grade squamous intraepithelial lesions, while CIN1 is considered a low-grade lesion.^5^ A 2011 study first suggested its effect post-cervical excision, while the 2012 FUTURE trial indicated reduced CIN2+ recurrence risk in previously vaccinated young women.^6^^,^^7^ By 2024, 21 studies had explored this topic, with a recent synthesis of 20 studies underscoring the importance of vaccination timing relative to conisation.^8^

Based on these findings, we conducted a large retrospective cohort study using data collected over the past 15 years from one of the central laboratories in the Czech Republic. Specifically, we aimed to determine the effect of HPV vaccination, focusing on its timing, onset, persistence, and relapse rate in relation to cone margin status.

The main objective of this study was to estimate the effect of HPV vaccination on CIN2+ recurrence, relative to the timing of vaccination. This was also investigated in women based on whether they had a positive or negative cone margin following excision, as women with a positive cone margin have been shown to be at higher risk of recurrence.^9^^,^^10^

Secondary analyses were conducted to evaluate these effects, either regardless of vaccination timing or for any dysplasia, including low- or indeterminate-grade (CIN1+).

## Methods

### Study design and setting

This retrospective cohort study used records of cytology, histology, and HPV genotyping from cervical smear, biopsy, or conisation samples processed at the central laboratory of UNILABS Pathology k.s., which operates five branches across the Czech Republic. Nearly 33% of Czech women aged 25–55 years undergoing national cervical cancer screening were examined in this laboratory.^11^ Each sample was submitted by a gynaecology clinic with a request form that included the year of the last HPV vaccination and the trade name of the vaccine. The laboratory provided records for women who had at least one cervical loop excision between 2010 and 2024, as recommended in the Czech Republic.^12^ Patients with multiple follow-up records after conisation were included, except for those with a history of hysterectomy or related procedures. The study was registered in the ClinicalTrials.gov international database (NCT06258564) and approved by the Ethics Committee of the Third Faculty of Medicine, Charles University, Prague, Czech Republic (26 February 2024).

### Data extracted from laboratory records

The year of cervical excision and the gynaecology clinic performing the procedure were considered potential confounders and were extracted from laboratory records. Gynaecology clinics were stratified based on the number of conisations (>500, 100–500, <100) to minimize the potential influence of differences between sites with high or low volumes of cervical excisions on CIN recurrence rates. The year of conisation was categorized into three 5-year periods (2010–2014, 2015–2019, and 2020–2024) to account for variations in the number of screened and vaccinated women over the 15-year follow-up reflecting the expansion of the central laboratory.

Histology findings from conisation or prior biopsy determined the CIN grade or carcinoma, while those without a grade were classified as undetermined dysplasia. The likely presence of HPV on the cervix was detected through p16 immunohistochemistry performed on samples from conisation or a prior biopsy.^13^ Additionally, cone margin positivity was determined based on the histologist’s report, with unspecified margins classified as unknown.

Women were classified as vaccinated prophylactically or post-excision if their last HPV vaccine dose was administered before or within one year of conisation, respectively. Post-excision vaccination was classified based on the timing of the last dose, regardless of whether the vaccination was initiated before or after the surgical excision. Those who received vaccination in the same year as repeat conisation or later were classified as unvaccinated. Women whose last reported vaccination occurred more than two years after conisation were excluded, as they may have started vaccination more than one year after excision. If the vaccination year was missing, the woman was classified as vaccinated regardless of timing for the purpose of sensitivity analyses.

The laboratory database lacked information on the completeness of vaccination. Therefore, ten outpatient clinics were consulted to estimate the proportion of women fully immunised after conisation. These clinics, located across different regions, were selected based on their ability to capture a diverse patient population. They were chosen to ensure a representative sample of women who underwent conisation, allowing for a reliable estimate of vaccination completion.

### Study endpoints

The primary endpoint was defined as the recurrence rate based on histologically confirmed CIN2+ from conisation or a previous biopsy specimen. This rate was related to the period from conisation to re-conisation in women with CIN relapse, and to the period from conisation to the last histologically or cytologically confirmed negative finding in women without relapse, during a follow-up of up to 15 years.

The influence of factors was examined in specific subgroups for both prior and post-excision HPV vaccinations. These factors included the type of the commercial vaccine used (2v-, 4v-, and 9vHPV vaccines) and age at the time of conisation, which was stratified into <30 years, 30–44 years, and >44 years based on the quartile distribution of age. This categorization reflected the fact that conisation was most frequently performed in women aged 30–44, with the <30 and >44 groups representing the lower and upper extremes of the age spectrum. Additionally, the factors included intervals of 60, 180, and 365 days between conisation and recurrence assessment, which have been applied in other similar studies. The use of these intervals also facilitates comparison with previously published data and enhances the interpretability of the findings within the context of existing literature. To evaluate the early, medium, and long-term effects of HPV vaccination, follow-up periods of 6 months, 18 months, 3 years, and 6 years after cervical excision were analysed. Additionally, the effect of prophylactic HPV vaccination on CIN recurrence was investigated in women vaccinated before the age of 18 and those vaccinated at 18 or older. This grouping reflects the fact that most women receive prophylactic vaccination during early adolescence as part of national immunisation programs, and that higher vaccine effectiveness has been documented in those vaccinated before the age of 18.^14^

### Statistical methods/analysis

Categorical variables were presented as absolute numbers and percentages, while continuous ones were reported as mean and standard deviation (SD). The Kaplan–Meier estimator was employed to determine the probability of CIN recurrence.

The crude incidence rate ratio (IRR), along with its 95% confidence interval (95% CI), was calculated based on the incidence rates of CIN in vaccinated versus unvaccinated women, with the rates expressed per 1000 person-years (py). Poisson regression was used to adjust the IRR for covariates representing potential confounding variables including stratified age at conisation, cone margin positivity, HPV detection, year of excision, and clinical site. The log-rank test was applied to assess the equality of failure functions. A goodness-of-fit statistic was used to evaluate the adequacy of the Poisson models. In cases where the model assumptions were not met, Cox proportional hazards regression was considered an appropriate alternative approach. Vaccine effectiveness, as a measure of reduction in recurrence, was estimated from the adjusted IRRs using the formula: 100% × (1–IRR).

A sensitivity analysis was conducted for women undergoing conisation for CIN1+ and for vaccinated women regardless of timing, including those with missing data on the year of vaccination. Subgroup analyses were performed post hoc based on the type of the commercial vaccine used, age at the time of conisation, length of the interval between conisation and recurrence assessment, age at prophylactic vaccination, and duration of follow-up.

To assess whether the study outcomes had sufficient statistical power to detect a meaningful difference in CIN recurrence rates between vaccinated and unvaccinated women, the power of the test was calculated. The log-rank test was used to compare two recurrence rates based on the Freedman method.

All tests were two-tailed, with the significance level set at 0·05. Statistical analyses and regressions were conducted using Prism 10 (GraphPad Software, Inc., San Diego, CA, USA) and STATA/SE version 18 (StataCorp. 2023. Stata Statistical Software: Release 18. College Station, TX: StataCorp LLC) software.

### Role of funding source

This work was supported by the Cooperatio 31 fund, Health Sciences, Charles University, Prague, Czech Republic. The funder played no role in the study design, data analysis, data interpretation, or the writing of this report.

## Results

### Characteristics of the study population

**Fig. 1:**
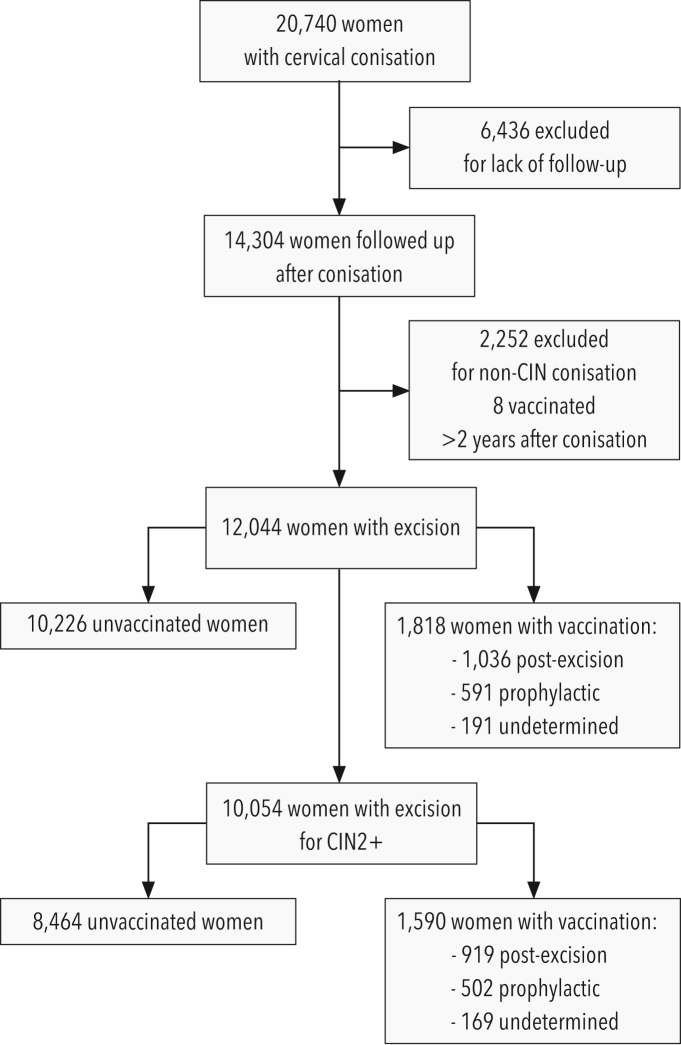
**Flowchart of cohort study.** CIN, cervical intraepithelial neoplasia any grade including indeterminate dysplasia and carcinoma. CIN2+, cervical intraepithelial neoplasia grade 2 or worse and carcinoma.

The cohort study analysed data extracted from records between January 2010 and December 2024. Of the 20,740 women undergoing cervical excision, 14,304 were followed up, and 12,044 had excision for any dysplasia. Among all 2045 vaccinated women, 63 were reclassified as unvaccinated due to immunisation occurring after re-excision, eight vaccinated more than two years after conisation were excluded, and 156 underwent conisation for a non-CIN reason. Fig. 1 shows the cohort flowchart.

Of 546 re-conisations for CIN2+, relapses were more frequent in unvaccinated women (6·1%) than in those vaccinated: 1·6% post-conisation, 2·8% prophylactically, and 2·4% regardless of timing (Table 1). The mean age at excision was 36·1 ± 9·5 years (±SD), 33·0 ± 6·9 years for women vaccinated after conisation, and 30·6 ± 6·5 years for those vaccinated before. Women vaccinated before conisation received the vaccine at 23·3 ± 7·1 years of age, with excision occurring 7·2 ± 4·0 years later. The mean follow-up after excision was 4·2 ± 3·4 years, with 4·9 ± 3·0 years for post-excision vaccinated women and 4·8 ± 3·3 years for those vaccinated prophylactically. The results of cone histology, including margin positivity, HPV detection, the type of the commercial HPV vaccine used, and stratification by clinic site and year of excision, are shown in Table 1 (Supplementary Table S1). Beyond the primary dataset, an additional survey conducted in 10 outpatient clinics revealed that 289 out of 303 women (95·4%) had been fully vaccinated with three doses after conisation.

**Table 1:** Baseline characteristics of women undergoing conisation for cervical intraepithelial neoplasia grade 2 or worse (CIN2+) and carcinoma, stratified by vaccination status, based on data from the study cohort collected between 2010 and 2024 in the UNILABS central laboratory.

Variable	All^a^	Unvaccinated	Vaccinated		
			Post-excision	Prophylactic	Undetermined
No. of conisations	10,054	8464	919	502	169
No. of re-conisations (%)	546 (5·4)	513 (6·1)	15 (1·6)	14 (2·8)	4 (2·4)
Mean age at conisation in years (SD)	36·1 (9·5)	36·9 (9·7)	33·0 (6·9)	30·6 (6·5)	33·4 (7·4)
Mean years of follow-up (SD)	4·2 (3·4)	4·2 (3·4)	4·9 (3·0)	4·8 (3·3)	4·2 (2·9)
Mean age at vaccination in years (SD)	30·1 (8·6)		33·5 (7·0)	23·3 (7·1)	
Age groups					
<30 years (%)	2677 (26·6)	2055 (24·3)	324 (35·3)	242 (48·2)	56 (33·1)
30–44 years (%)	5623 (55·9)	4747 (56·1)	529 (57·6)	245 (48·8)	102 (60·4)
>44 years (%)	1754 (17·4)	1662 (19·6)	66 (7·2)	15 (3·0)	11 (6·5)
No. of women with conisation for					
CIN2 (%)	3627 (36·1)	3069 (36·3)	283 (30·8)	216 (43·0)	59 (34·9)
CIN3 (%)	6005 (59·7)	5021 (59·3)	605 (65·8)	276 (55·0)	103 (60·9)
Carcinoma (%)	422 (4·2)	374 (4·4)	31 (3·4)	10 (2·0)	7 (4·1)
No. of women with re-conisation for					
CIN2 (%)	151 (27·7)	143 (27·9)	3 (20·0)	5 (35·7)	0 (0·0)
CIN3 (%)	336 (61·5)	312 (60·8)	12 (80·0)	8 (57·1)	4 (100·0)
Carcinoma (%)	59 (10·8)	58 (11·3)	0 (0·0)	1 (7·1)	0 (0·0)
No. of women with cone margin at conisation					
Positive (%)	1796 (17·9)	1568 (18·5)	119 (12·9)	84 (16·7)	25 (14·8)
Negative (%)	7268 (72·3)	6032 (71·3)	728 (79·2)	381 (75·9)	127 (75·1)
Unknown (%)	990 (9·8)	864 (10·2)	72 (7·8)	37 (7·4)	17 (10·1)
No. of women with cone margin at re-conisation					
Positive (%)	139 (25·5)	128 (25·0)	4 (26·7)	5 (35·7)	2 (50·0)
Negative (%)	326 (59·7)	306 (59·6)	11 (73·3)	7 (50·0)	2 (50·0)
Unknown (%)	81 (14·8)	79 (15·4)	0 (0·0)	2 (14·3)	0 (0·0)
No. of women with HPV detected at conisation					
No (%)	180 (1·8)	157 (1·9)	11 (1·2)	10 (2·0)	2 (1·2)
Yes (%)	5673 (56·4)	4675 (55·2)	585 (63·7)	312 (62·2)	101 (59·8)
Unknown (%)	3673 (36·5)	3119 (36·9)	308 (33·5)	180 (35·9)	66 (39·1)
No. of women with HPV detected at re-conisation					
No (%)	44 (8·1)	37 (7·2)	2 (13·3)	3 (21·4)	2 (50·0)
Yes (%)	275 (50·4)	258 (50·3)	11 (73·3)	4 (28·6)	2 (50·0)
Unknown (%)	227 (41·6)	218 (42·5)	2 (13·3)	7 (50·0)	0 (0·0)
No. of women with vaccine type					
2vHPV (%)	288 (2·9)		112 (12·2)	151 (30·1)	25 (14·8)
4vHPV (%)	531 (5·3)		186 (20·2)	305 (60·8)	40 (23·7)
9vHPV (%)	718 (7·1)		611 (66·5)	36 (7·2)	71 (42·0)
Unknown (%)	53 (0·5)		10 (1·1)	10 (2·0)	33 (19·5)
No. of women at gynaecology clinic with					
>500 excisions (%)	7873 (78·3)	6529 (77·1)	760 (82·7)	449 (89·4)	135 (79·9)
100–500 excisions (%)	1791 (17·8)	1569 (18·5)	144 (15·7)	46 (9·2)	32 (18·9)
<100 excisions (%)	390 (3·9)	366 (4·3)	15 (1·6)	7 (1·4)	2 (1·2)
No. of women by year of conisation					
2010–2014 (%)	2001 (19·9)	1759 (20·8)	130 (14·1)	89 (17·7)	23 (13·6)
2015–2019 (%)	3976 (39·5)	3261 (38·5)	433 (47·1)	207 (41·2)	75 (44·4)
2020–2024 (%)	4077 (40·6)	3444 (40·7)	356 (38·7)	206 (41·0)	71 (42·0)

SD, standard deviation. 2vHPV, bivalent HPV vaccine. 4vHPV, quadrivalent HPV vaccine. 9vHPV, nonavalent PV vaccine. CIN2, cervical intraepithelial neoplasia grade 2. CIN3, cervical intraepithelial neoplasia grade 3.

a Percentages may not total 100% because of rounding.

### Outcomes of the primary analysis

During the follow-up period of up to 15 years, the CIN2+ recurrence rate was 14·61 per 1000 py in unvaccinated women, decreasing to 3·37 in women vaccinated post-excision, and 5·84 in those vaccinated prophylactically. HPV vaccination reduced recurrence risk by 74% (95% CI: 57–85%) when completed after conisation, and 54% (95% CI: 22–73%) when administered prophylactically (Table 2). Both results had >80% statistical power, indicating the sample size was sufficient. Although the recurrence rate was higher in prophylactically vaccinated women compared with those vaccinated post-excision, the difference was not statistically significant, as indicated by an aIRR of 1·77 (95% CI: 0·85–3·67; p = 0·125) (see Supplementary Fig. S1).

**Table 2:** Recurrence rates of cervical intraepithelial neoplasia grade 2 or worse (CIN2+) and carcinoma by timing of HPV vaccination and cone margin positivity.

HPV vaccination status	No. of recurrences	Person-years (py)	Incidence rate per 1000 py (95% CI)	Crude incidence rate ratio (95% CI)	Adjusted incidence rate ratio (95% CI)^a^	Vaccine effectiveness (95% CI)
Unvaccinated	513	35,105	14·61 (13·4–15·93)	Reference	Reference	Reference
Vaccinated irrespective of timing	33	7565	4·36 (3·1–6·14)	0·3 (0·2–0·42)	0·33 (0·23–0·47)	67 (53–77)^b^
Vaccinated post-excision	15	4455	3·37 (2·03–5·58)	0·23 (0·13–0·38)	0·26 (0·15–0·43)	74 (57–85)^b^
Vaccinated prophylactically	14	2397	5·84 (3·46–9·86)	0·4 (0·22–0·68)	0·46 (0·27–0·78)	54 (22–73)^b^
Women with positive cone margin						
Unvaccinated	272	5269	51·62 (45·84–58·14)	Reference	Reference	Reference
Vaccinated irrespective of timing	13	1129	11·51 (6·68–19·82)	0·22 (0·12–0·39)	0·26 (0·15–0·46)	74 (54–85)^b^
Vaccinated post-excision	6	613	9·78 (4·4–21·78)	0·19 (0·07–0·42)	0·21 (0·1–0·48)	79 (52–90)^b^
Vaccinated prophylactically	6	401	14·94 (6·71–33·26)	0·29 (0·11–0·64)	0·38 (0·17–0·86)	62 (14–83)
Women with negative cone margin						
Unvaccinated	187	25,945	7·21 (6·25–8·32)	Reference	Reference	Reference
Vaccinated irrespective of timing	17	5838	2·91 (1·81–4·68)	0·4 (0·23–0·66)	0·42 (0·26–0·7)	58 (30–74)^b^
Vaccinated post-excision	7	3494	2 (0·96–4·2)	0·28 (0·11–0·58)	0·29 (0·13–0·61)	71 (39–87)^b^
Vaccinated prophylactically	7	1822	3·84 (1·83–8·06)	0·53 (0·21–1·12)	0·59 (0·27–1·26)	41 (−26 to 73)

a Poisson regression models adjusted for age groups, cone margin positivity (full analysis set), and HPV detection, with stratification by year and gynaecology clinic of conisation.

b Result with a p < 0·05 at statistical power of >80%.

A higher risk of CIN2+ recurrence was observed in women with a positive cone margin compared to those with a negative margin, as shown by an aIRR of 7·21 (95% CI: 6·02–8·63) (see Supplementary Fig. S1). In unvaccinated women with positive cone margins, recurrence risk reached 51·62 per 1000 py. Post-conisation vaccination reduced the risk to 9·78 per 1000 py, representing a 79% reduction (95% CI: 52–90%) with >90% statistical power. In contrast, prophylactic vaccination was associated with a 62% reduction (95% CI: 14–83%), lowering the rate to 14·94 per 1000 py but lacking statistical power.

**Fig. 2:**
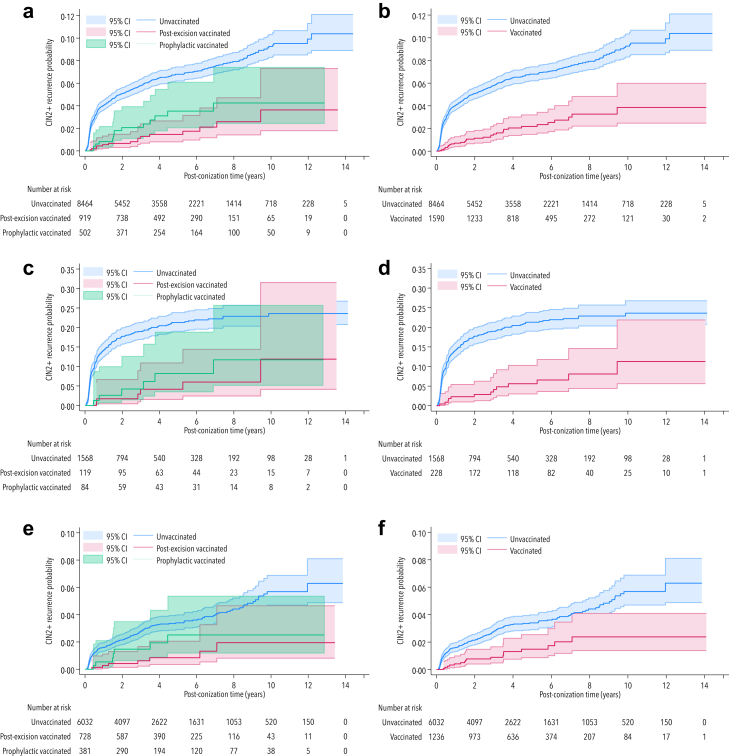
**Probability of recurrence of cervical intraepithelial neoplasia grade 2 or worse (CIN2+) and carcinoma in HPV-vaccinated and unvaccinated women, relative to time since conisation: a) prophylactic and post-excision vaccination, in women regardless of cone margin status, b) regardless of vaccination timing and cone margin status, c) prophylactic and post-excision vaccination in women with positive cone margins, d) regardless of vaccination timing, in women with positive cone margins, e) prophylactic and post-excision vaccination in women with negative cone margins, f) regardless of vaccination timing in women with negative cone margins.** 95% CI, 95% confidence interval.

Among unvaccinated women with negative cone margins, the recurrence rate was 7·21 per 1000 py, while post-excision vaccination significantly reduced the rate to 2·00 (71% effect, 95% CI: 39–87%). Prophylactic vaccination lowered the rate to 3·84 per 1000 py, but the 41% reduction (95% CI: −26 to 73%) was not statistically significant. These results are shown in the Kaplan–Meier curve (Fig. 2).

### Outcomes of the secondary analyses

The recurrence rates of any dysplasia were 15·12 per 1000 py in unvaccinated women, 3·79 in those vaccinated after conisation, and 6·46 in those previously vaccinated (Supplementary Table S2). Compared with unvaccinated women, recurrence rates were reduced by 73% (95% CI: 57–83%) following post-conisation vaccination and by 54% (95% CI: 27–71%) for prophylactic one, both with >80% statistical power. These findings align with those of the primary analysis and confirm consistent vaccine effects on CIN1+ recurrence regardless of cone margin status.

An analysis independent of HPV vaccination timing, including women with unknown vaccination year, confirmed reductions of 67% reduction (95% CI: 53–77%) in CIN2+ recurrence and of 65% (95% CI: 52–74%) in any dysplasia recurrence (Table 2). The effect was stronger with positive cone margins, reducing CIN2+ recurrence by 74% (95% CI: 54–85%) versus 58% (95% CI: 30–74%) with negative cone margins. Fig. 2 shows the probability of CIN2+ recurrence over time.

### Outcomes of the subgroup analyses

#### Type of HPV vaccine

The highest vaccination effect on CIN2+ recurrence, 83% (95% CI: 61–92%), was observed with the 9vHPV vaccine administered after conisation (Table 3). Post-conisation, 2vHPV vaccine reduced recurrence by 56% (95% CI: −18 to 84%), but this reduction was not statistically significant. In contrast, 4vHPV vaccine showed a statistically significant 64% reduction (95% CI: 13–85%), although its statistical power of 57% may have been limited by the sample size. Reductions of 66% and 42% were not significant with prophylactic vaccination using the 2vHPV and 4vHPV, respectively. Only 36 women received 9vHPV vaccination before conisation, with no relapses, making an effect estimate impossible.

**Table 3:** Recurrence rates of cervical intraepithelial neoplasia grade 2 or worse (CIN2+) and carcinoma by timing of HPV vaccination relative to vaccine type, age group at conisation, and stratified follow-up after excision.

HPV vaccination status	No. of recurrences	Person-years (py)	Incidence rate per 1000 py (95% CI)	Crude incidence rate ratio (95% CI)	Adjusted incidence rate ratio (95% CI)^a^	Vaccine effectiveness (95% CI)
Unvaccinated	513	35,105	14·61 (13·4–15·93)	Reference	Reference	Reference
2vHPV vaccinated post-excision	4	830	4·82 (1·81–12·83)	0·33 (0·09–0·85)	0·44 (0·16–1·18)	56 (−18 to 84)
2vHPV vaccinated prophylactically	3	749	4·01 (1·29–12·42)	0·27 (0·06–0·81)	0·34 (0·11–1·07)	66 (−7 to 89)
4vHPV vaccinated post-excision	5	1322	3·78 (1·57–9·09)	0·26 (0·08–0·61)	0·36 (0·15–0·87)	64 (13–85)
4vHPV vaccinated prophylactically	11	1482	7·42 (4·11–13·4)	0·51 (0·25–0·92)	0·58 (0·32–1·06)	42 (−6 to 68)
9vHPV vaccinated post-excision	6	2264	2·65 (1·19–5·9)	0·18 (0·07–0·4)	0·17 (0·08–0·39)	83 (61–92)^b^
9vHPV vaccinated prophylactically	0	123				
Women under 30 years at conisation						
Unvaccinated	91	10,008	9·09 (7·4–11·17)	Reference	Reference	Reference
Vaccinated post-excision	6	1780	3·37 (1·51–7·5)	0·37 (0·13–0·84)	0·35 (0·15–0·81)	65 (19–85)
Vaccinated prophylactically	3	1314	2·28 (0·74–7·08)	0·25 (0·05–0·76)	0·24 (0·08–0·77)	76 (23–92)
Women aged 30–44 years at conisation						
Unvaccinated	280	19,918	14·06 (12·5–15·8)	Reference	Reference	Reference
Vaccinated post-excision	9	2459	3·66 (1·9–7·03)	0·26 (0·12–0·5)	0·25 (0·13–0·49)	75 (51–87)^b^
Vaccinated prophylactically	11	1020	10·78 (5·97–19·46)	0·77 (0·38–1·39)	0·63 (0·35–1·16)	37 (−16 to 65)
Women over 44 years at conisation						
Unvaccinated	142	5179	27·42 (23·26–32·32)	Reference	Reference	Reference
Vaccinated post-excision	0	216				
Vaccinated prophylactically	0	62				
Within 6 months of follow-up post-excision						
Unvaccinated	235	4011	58·59 (51·56–66·58)	Reference	Reference	Reference
Vaccinated post-excision	2	452	4·42 (1·11–17·69)	0·08 (0·01–0·28)	0·11 (0·03–0·43)	89 (57–97)^b^
Vaccinated prophylactically	2	245	8·16 (2·04–32·61)	0·12 (0·02–0·46)	0·2 (0·05–0·81)	80 (19–95)
Within 18 months of follow-up post-excision						
Unvaccinated	367	10,914	33·63 (30·36–37·25)	Reference	Reference	Reference
Vaccinated post-excision	6	1345	4·46 (2–9·93)	0·13 (0·05–0·29)	0·17 (0·08–0·38)	83 (62–92)^b^
Vaccinated prophylactically	5	701	7·13 (2·97–17·13)	0·2 (0·06–0·47)	0·27 (0·11–0·66)	73 (34–89)^b^
Within 3 years of follow-up post-excision						
Unvaccinated	430	18,691	23·01 (20·93–25·29)	Reference	Reference	Reference
Vaccinated post-excision	10	2414	4·14 (2·23–7·7)	0·18 (0·09–0·33)	0·22 (0·12–0·41)	78 (59–88)^b^
Vaccinated prophylactically	9	1239	7·26 (3·78–13·96)	0·31 (0·14–0·59)	0·39 (0·2–0·75)	61 (25–80)^b^
Within 6 years of follow-up post-excision						
Unvaccinated	477	28,341	16·83 (15·39–18·41)	Reference	Reference	Reference
Vaccinated post-excision	12	3727	3·22 (1·83–5·67)	0·19 (0·1–0·34)	0·23 (0·13–0·4)	77 (60–87)^b^
Vaccinated prophylactically	13	1928	6·74 (3·91–11·61)	0·4 (0·21–0·69)	0·48 (0·27–0·83)	52 (17–73)
60-day interval since excision						
Unvaccinated	472	35,090	13·45 (12·29–14·72)	Reference	Reference	Reference
Vaccinated post-excision	15	4455	3·37 (2·03–5·58)	0·25 (0·14–0·42)	0·28 (0·17–0·46)	72 (54–83)^b^
Vaccinated prophylactically	14	2397	5·84 (3·46–9·86)	0·43 (0·24–0·74)	0·49 (0·29–0·84)	51 (16–71)
180-day interval since excision						
Unvaccinated	278	34,895	7·97 (7·08–8·96)	Reference	Reference	Reference
Vaccinated post-excision	13	4453	2·92 (1·7–5·03)	0·37 (0·19–0·64)	0·37 (0·21–0·65)	63 (35–79)^b^
Vaccinated prophylactically	12	2392	5·02 (2·85–8·83)	0·63 (0·32–1·12)	0·63 (0·35–1·12)	37 (−12 to 65)
365-day interval since excision						
Unvaccinated	186	34,224	5·43 (4·71–6·27)	Reference	Reference	Reference
Vaccinated post-excision	10	4436	2·25 (1·21–4·19)	0·41 (0·2–0·78)	0·41 (0·21–0·77)	59 (23–79)^b^
Vaccinated prophylactically	10	2368	4·22 (2·27–7·85)	0·78 (0·37–1·46)	0·73 (0·38–1·38)	27 (−38 to 62)

a Poisson regression models adjusted for age group (except in age analyses), cone margin positivity, and HPV detection, with stratification by year and gynaecology clinic of conisation.

b Result with a p < 0·05 at statistical power of >80%.

#### Age of women at conisation

Regardless of vaccination, the risk of relapse was lower in women younger than 30 years compared to those aged 30–44 years (aIRR = 0·68; 95% CI: 0·54–0·86), and significantly higher in women older than 45 years (aIRR = 1·58; 95% CI: 1·29–1·93) (Supplementary Fig. S1). In unvaccinated women, the CIN2+ recurrence rate per 1000 py increased with age at conisation, from 9·09 in those under 30 to 14·06 in the 30–44 age group and 27·42 in women 45 and older (Table 3). Among post-excision vaccinated women, the rate remained stable at 3·37–3·66 per 1000 py across all age groups, with effects in 65% (95% CI: 19–85%) in women under 30, and 75% (95% CI: 51–87%) in those aged 30–44. No relapses were observed in 66 women vaccinated at age 45 and older.

Previously vaccinated women undergoing excision before age 30 had a recurrence rate of 2·28 per 1000 py, compared with 10·78 in those aged 30–44. A 76% reduction (95% CI: 23–92%) was significant only in younger women. No relapses were observed in 15 previously vaccinated women aged >44, thus precluding analysis.

#### Follow-up duration after excision

The persistence of HPV vaccination effect was assessed over four follow-up periods (Table 3). In unvaccinated women, CIN2+ recurrence rates per 1000 py fell from 58·59 at 6 months to 33·63 at 18 months, 23·01 at 3 years, and 16·83 at 6 years. The rates declined from 4·42 to 3·22 in women vaccinated post-excision and from 8·16 to 6·74 in those vaccinated prior to conisation over a follow-up period of 6 months to 6 years.

The greatest reductions in recurrence were seen within the first 6 months, 89% in post-excision vaccinated women, and 80% in prophylactically vaccinated women. By 3 and 6 years, post-conisation vaccination reduced relapses by 78% and 77%, respectively, compared to 61% and 52% with prophylactic vaccination.

#### Age at prophylactic HPV vaccination

The effect was greater but not statistically significant in women vaccinated at or before 18 years of age, with an 85% reduction in CIN2+ relapses (95% CI: −5 to 98%) compared to 45% (95% CI: 5–69%) in those vaccinated after 18 years (Supplementary Table S3). However, the recurrence rate was lower at 1·81 per 1000 py in the former compared with 7·05 in the latter.

#### Interval between conisation and follow-up

The effect of HPV vaccination diminished with longer relapse assessment intervals of 60, 180, and 365 days following conisation (Table 3). While CIN2+ recurrence rates markedly decreased from 13·45 per 1000 py at the 60-day interval to 5·43 at the 365-day interval in unvaccinated women, the rates declined more gradually in vaccinated women, from 3·37 to 2·25 in those vaccinated post-excision, and from 5·84 to 4·22 in those vaccinated prior to conisation. Significant reductions of 63% after 180 days and 59% after 365 days was observed only in women vaccinated post-excision.

## Discussion

HPV vaccination reduced the risk of CIN2+ recurrence by 67% and any dysplasia by 65%, regardless of timing. This aligns with a pooled vaccine effectiveness of 70% (95% CI: 55–80%).^8^ The effect of vaccination as an adjuvant intervention was stronger in women vaccinated post-excision compared to those vaccinated earlier as part of preventive immunisation. The 74% reduction in CIN2+ recurrence in this study aligns with the 67–85% prediction interval from 12 prior studies on post-conisation vaccination.^8^ The lower 54% effect for prophylactic vaccination is consistent with the 50% reduction pooled from four earlier studies.^7^^,^^15^, ^16^, ^17^

Previous studies reported a more than fourfold increase in CIN2+ recurrence risk for women with positive cone margins compared with negative ones, but the impact of vaccination in this high-risk group has not been investigated to date.^9^^,^^10^ The present study demonstrates that women with positive cone margins are at high risk of CIN2+ recurrence, with a rate of 51·62 per 1000 py, reduced by 74% with HPV vaccination, regardless of timing. Post-excision vaccination decreased recurrence by 79% in high-risk women, having a rate of 9·78 per 1000 py, comparable to the 7·21 observed in unvaccinated women with negative cone margins. Vaccination after conisation reduced relapses by an additional 71% in women with negative cone margins, resulting in a rate of 2·00 per 1000 py. Previous HPV vaccination was more effective in women with a positive cone margin, lowering their risk of recurrence by 62%, compared with a non-significant 41% decline in those with a negative one. These results underscore the importance of post-excision vaccination, particularly for high-risk women with positive cone margins, while reaffirming the enduring value of prophylactic vaccination during adolescence or early adulthood.

The type of the HPV vaccine seems to be crucial since the greatest reduction in CIN2+ recurrence (83%) was observed in women receiving the 9vHPV vaccine after excision, with conclusive statistical power. This aligns with the 71–90% decline in CIN2+ recurrence reported in studies where most women received this vaccine.^9^^,^^18^^,^^19^ Likewise, a 64% reduction was noted with the 4vHPV vaccine post-conisation, matching the 59·4–78·3% prediction interval from a recent meta-analysis.^8^ Unfortunately, while the 2vHPV vaccine showed a 56–66% decrease in recurrence, the results lacked statistical proof. Post-excision 2vHPV vaccination has not yet been evaluated separately in previous studies. When used prophylactically, it showed either a substantial reduction in CIN2+ recurrence (88%) or an uncertain effect, with 95% confidence intervals ranging from negative values to 84%.^15^, ^16^, ^17^

In unvaccinated women, the CIN2+ recurrence rate increased with advancing age at conisation. Post-excision vaccination reduced relapses by 65–75% in women undergoing excision under 45 years of age. However, a similar 76% effect was observed in women prophylactically vaccinated and undergoing conisation before the age 30.

Surprisingly, a strong effect was observed within the first six months of conisation in women with both incomplete and complete HPV vaccination. Prophylactic vaccination reduced recurrence by 80%, and post-excision by 89%, compared with the rate of 58·59 per 1000 py in unvaccinated women. Since the recurrence rates did not increase and continued to slightly decline 1·2–1·4-fold over 1·5–6 years in HPV-vaccinated women, it can be presumed that the long-term benefit of HPV vaccination extends for at least six years, regardless of vaccination timing. Furthermore, within six years of follow-up after conisation, relapse was reduced by 77% in women vaccinated post-excision and by 52% in prophylactically vaccinated women. While such long-term follow-up has not yet been studied in prophylactically vaccinated women, the effect of post-excision vaccination was comparable with the 79–89% reduction reported in studies with five-to six-year follow-up periods.^19^^,^^20^ We believe the agreement between new and existing results supports the estimation of at least six years of persistence for the effect of HPV vaccination, especially when administered after conisation.

Most studies assessed recurrence at 2–3 months or 4–7 months after conisation, while one study evaluated it as early as 1 month and another one as late as 1 year after conisation.^7^^,^^10^^,^^15^^,^^17^^,^^18^^,^^21^, ^22^, ^23^, ^24^, ^25^, ^26^, ^27^, ^28^ To address this variability, we examined CIN2+ recurrence at 2, 6, and 12 months post-conisation. With the interval between conisation and relapse evaluation increasing from 2 to 12 months, a substantial 2·5-fold decline in recurrence rates was observed in unvaccinated women, compared with a more modest approximately 1·5-fold, decline in HPV-vaccinated women. Consequently, this naturally resulted in a diminishing effect of HPV vaccination, regardless of its timing. This may have contributed to the non-significant 14% reduction in CIN2+ relapses assessed one year after conisation, as observed in women with HPV vaccination completed post-excision in a Danish study from 2019.^28^ Whether this finding was associated with the delayed timing of assessment or with other study-specific factors—such as the inclusion of only women with CIN3-related conisation or unreported cone margin status—cannot be clearly determined. Nevertheless, we believe that assessing early recurrence represents a novel approach contributing to a better understanding of HPV vaccination benefits.

An interesting finding was the early onset of effect in women completing vaccination either before or after conisation. This suggests that adaptive immunity, whether induced by a recent partial vaccination or persisting from prior immunisation, may contribute equally to the protective effect. This aligns with results of a study confirming HPV clearance in women vaccinated within the first year of conisation.^29^

HPV infection appears to interact with the epithelial microenvironment, stimulating pro-inflammatory cytokines.^30^ Furthermore, the precancerous environment suppresses antiviral responses and activates multiple immunosuppressive factors, thus contributing to immune escape.^31^ Surgical excision of cervical neoplasia likely reduces cytokine levels, removes the precancerous microenvironment, and triggers an anti-inflammatory response as part of the healing process.^32^ This process likely enhances the onset of adaptive immunity acquired through past or current HPV vaccination, as suggested by recent study findings.^33^

Our study has several notable strengths. As a large-scale, population-based investigation, it provides sufficient statistical power to estimate the effects of HPV vaccination, particularly regarding cone margin positivity and vaccination timing. With a follow-up period of up to 15 years, it comprehensively evaluates the long-term persistence of the vaccination effect. We collected data on potential confounders including attained age, calendar year, clinical site of conisation, cone margin positivity, and HPV presence. Adjusting for these factors minimized their confounding effects, and sensitivity analyses confirmed the robustness of our estimates. Subgroup analyses further explored potential biases related to vaccine type, age at conisation, and intervals between conisation and recurrence assessment.

However, certain limitations should be noted. The first was the lack of detailed information on complete vaccination, particularly regarding the administration of three doses post-conisation and whether two or three doses were given prophylactically. This limitation was partially mitigated through a supplementary survey at outpatient clinics, which confirmed full vaccination in most immunised women. Owing to the lack of HPV genotyping prior to conisation, it was not possible to determine whether the observed effect on recurrence was HPV type-specific. This also limited a direct comparison between prophylactic and post-excision vaccination, as more than 90% of women in the prophylactic group received a bi- or quadrivalent HPV vaccine, while over 66% of women were immunised with the nonavalent vaccine after excision. Furthermore, it was not possible to explore whether the effect may have been influenced by the timing of vaccination initiation post-conisation, as exact dates were not available. The exclusion of women with an absent year of vaccination or last vaccination more than two years after conisation likely did not affect the results of this study, as documented by the sensitivity analysis and an acceptable loss of vaccination year data in fewer than 12% of women. Missing data on cone margin positivity or HPV detection were categorized as unknown. Furthermore, factors such as comorbidities, nulliparity, smoking, body mass index, and deprivation index were not included. Despite these omissions, we believe that incorporating age, cone margin positivity, HPV detection, and stratification by year of conisation and clinical site were essential to achieving the study objectives.

The study demonstrated an early onset of the effect of HPV vaccination in women undergoing cervical excision irrespective of whether vaccination was completed pre- or post-conisation. A particularly notable finding was the significant reduction in recurrence rates among women with a positive cone margin, highlighting the potential benefits of targeted HPV vaccination for this high-risk group.

The lower effectiveness of prophylactic vaccination raises the question of whether a booster dose administered post-conisation could enhance its effect. Additionally, the early strong protective effect of incomplete post-excision vaccination observed within the first six months suggests that similar benefits might be achievable with just one or two doses, as seen with prophylactic vaccination in individuals under 20 years of age.^2^^,^^34^

In conclusion, HPV vaccination significantly reduced CIN2+ recurrence in women undergoing surgical excision, with the greatest impact observed in those with positive cone margins. Although post-excision vaccination showed a tendency toward a greater effect than prophylactic vaccination, HPV immunisation overall provided meaningful benefits, particularly within the first six months of conisation. Additionally, the stable recurrence rates observed among vaccinated women throughout the study period support the favourable long-term persistence of this effect.

## Contributors

MP, MT, VD, PD, and JR conceptualized the study. MP, PD, and JR designed the study. MP, DL, and IKL curated the data. IF, MT and VDjr accessed and verified the underlying study data. VD and VDjr contributed to data interpretation. MP did the formal data analysis. MP, JM, DL, IKL, and PD contributed to the methodology. PD and JR ensured funding acquisition. MP and IKL administered the project. MP supervised the project. MP contributed to investigation, provision of computing resources, and visualisation. MP and IKL wrote the original draft, and all authors contributed to data interpretation and writing, reviewing, and editing this manuscript.

## Data sharing statement

The raw datasets are not available for sharing because of privacy policies and regulations in the Czech Republic. The data that support the findings of this study are available from the corresponding author upon reasonable request.

## Declaration of interests

We declare no competing interests.
