# Supplementary material for: Early and long-term effects of prophylactic and post-excision human papillomavirus vaccination on recurrent high-grade cervical intraepithelial neoplasia relative to margin status: a retrospective cohort study in the Czech Republic
Source: Lancet Reg Health Eur. 2025 Jun 3;55:101337. doi: 10.1016/j.lanepe.2025.101337 (PMC12167485; doi:10.1016/j.lanepe.2025.101337)
Supplement: Supplementary Fig. S1 and Tables S1–S3 [file mmc1.docx]

**Supplement**

**Table of content**

S-Table 1. Characteristics of study population including conisation for any dysplasia and carcinoma

S-Table 2. Recurrence rates of cervical intraepithelial neoplasia grade 1 or worse (CIN1+), carcinoma, and indeterminate dysplasia by timing of HPV vaccination and cone margin positivity

S-Table 3. Recurrence rates of cervical intraepithelial neoplasia grade 2 or worse (CIN2+) and carcinoma by age at vaccination before excision

S-Figure 1. Forest plot of adjusted incidence rate ratios (aIRR) with 95% confidence intervals (CI) and corresponding incidence rates (IR) for study covariates. A – Recurrence of cervical intraepithelial neoplasia grade 2 or worse (CIN2+) and carcinoma; B – Recurrence of any dysplasia and carcinoma.

S-Table 1. Characteristics of study population including conisation for any dysplasia and carcinoma

| **Variables** | **All^a^** | **Unvaccinated** | **Vaccinated** |  |  |
| --- | --- | --- | --- | --- | --- |
|  |  |  | **Post-excision** | **Prophylactic** | **Undetermined** |
| **No. of conisations** | 12,044 | 10226 | 1036 | 591 | 191 |
| **No. of re-conisations (%)** | 698 (5·8) | 655 (6·4) | 19 (1·8) | 18 (3·0) | 6 (3·1) |
| **Mean age at conisation in years (SD)** | 36·8 (9·7) | 37·5 (9·9) | 33·4 (7·1) | 30·7 (6·7) | 33·5 (7·6) |
| **Mean years of follow-up (SD)** | 4·3 (3·4) | 4·2 (3·5) | 4·8 (3·0) | 4·7 (3·3) | 4·4 (3·1) |
| **Mean age at vaccination in years (SD)** | 30·4 (8·8) |  | 33·9 (7·2) | 23·5 (7·2) |  |
| **Age groups** |  |  |  |  |  |
| **<30 years (%)** | 3011 (25·0) | 2318 (22·7) | 349 (33·7) | 282 (47·7) | 62 (32·5) |
| **30–44 years (%)** | 6635 (55·1) | 5636 (55·1) | 601 (58·0) | 285 (48·2) | 113 (59·2) |
| **>44 years (%)** | 2398 (19·9) | 2272 (22·2) | 86 (8·3) | 24 (4·1) | 16 (8·4) |
| **No. of women with conisation for** |  |  |  |  |  |
| **CIN1 (%)** | 1936 (16·1) | 1710 (16·7) | 117 (11·3) | 87 (14·7) | 22 (11·5) |
| **CIN2 (%)** | 3627 (30·1) | 3069 (30·0) | 283 (27·3) | 216 (36·5) | 59 (30·9) |
| **CIN3 (%)** | 6005 (49·9) | 5021 (49·1) | 605 (58·4) | 276 (46·7) | 103 (53·9) |
| **Carcinoma (%)** | 422 (3·5) | 374 (3·7) | 31 (3·0) | 10 (1·7) | 7 (3·7) |
| **Indeterminate dysplasia** | 54 (0·4) | 52 (0·5) | 0 (0·0) | 2 (0·3) | 0 (0·0) |
| **No. of women with re-conisation for** | 123 (17·6) | 114 (17·4) | 3 (15·8) | 4 (22·2) | 2 (33·3) |
| **CIN1 (%)** | 160 (22·9) | 152 (23·2) | 3 (15·8) | 5 (27·8) | 0 (0·0) |
| **CIN2 (%)** | 346 (49·6) | 322 (49·2) | 12 (63·2) | 8 (44·4) | 4 (66·7) |
| **CIN3 (%)** | 62 (8·9) | 61 (9·3) | 0 (0·0) | 1 (5·6) | 0 (0·0) |
| **Carcinoma (%)** | 7 (1·0) | 6 (0·9) | 1 (5·3) | 0 (0·0) | 0 (0·0) |
| **Indeterminate dysplasia** | 123 (17·6) | 114 (17·4) | 3 (15·8) | 4 (22·2) | 2 (33·3) |
| **No. of women with cone margin at conisation** |  |  |  |  |  |
| **Positive (%)** | 1985 (16·5) | 1732 (16·9) | 129 (12·5) | 97 (16·4) | 27 (14·1) |
| **Negative (%)** | 8662 (71·9) | 7261 (71·0) | 814 (78·6) | 446 (75·5) | 141 (73·8) |
| **Unknown (%)** | 1397 (11·6) | 1233 (12·1) | 93 (9·0) | 48 (8·1) | 23 (12·0) |
| **No. of women with cone margin at re-conisation** |  |  |  |  |  |
| **Positive (%)** | 157 (22·5) | 149 (22·7) | 4 (21·1) | 4 (22·2) | 0 (0·0) |
| **Negative (%)** | 413 (59·2) | 391 (59·7) | 13 (68·4) | 7 (38·9) | 2 (33·3) |
| **Unknown (%)** | 128 (18·3) | 115 (17·6) | 2 (10·5) | 7 (38·9) | 4 (66·7) |
| **No. of women with HPV detection at conisation** |  |  |  |  |  |
| **No (%)** | 310 (2·6) | 284 (2·8) | 11 (1·1) | 13 (2·2) | 2 (1·0) |
| **Yes (%)** | 7023 (58·3) | 5890 (57·6) | 654 (63·1) | 364 (61·6) | 115 (60·2) |
| **Unknown (%)** | 4711 (39·1) | 4052 (39·6) | 371 (35·8) | 214 (36·2) | 74 (38·7) |
| **No. of women with HPV detection at re-conisation** |  |  |  |  |  |
| **No (%)** | 64 (9·2) | 58 (8·9) | 2 (10·5) | 4 (22·2) | 0 (0·0) |
| **Yes (%)** | 363 (52·0) | 338 (51·6) | 15 (78·9) | 7 (38·9) | 3 (50·0) |
| **Unknown (%)** | 271 (38·8) | 259 (39·5) | 2 (10·5) | 7 (38·9) | 3 (50·0) |
| **No. of women with vaccine type** |  |  |  |  |  |
| **2vHPV (%)** | 352 (2·9) |  | 135 (13·0) | 185 (31·3) | 32 (16·8) |
| **4vHPV (%)** | 594 (4·9) |  | 216 (20·8) | 331 (56·0) | 47 (24·6) |
| **9vHPV (%)** | 791 (6·6) |  | 671 (64·8) | 45 (7·6) | 75 (39·3) |
| **Unknown (%)** | 63 (0·5) |  | 14 (1·4) | 12 (2·0) | 37 (19·4) |
| **No. of women at clinical site with** |  |  |  |  |  |
| **>500 excisions (%)** | 9357 (77·7) | 7837 (76·6) | 846 (81·7) | 525 (88·8) | 149 (78·0) |
| **100–500 excisions (%)** | 2175 (18·1) | 1908 (18·7) | 169 (16·3) | 58 (9·8) | 40 (20·9) |
| **<100 excisions (%)** | 512 (4·3) | 481 (4·7) | 21 (2·0) | 8 (1·4) | 2 (1·0) |
| **No. of women by year of conisation** |  |  |  |  |  |
| **2010–2014 (%)** | 2503 (20·8) | 2210 (21·6) | 154 (14·9) | 109 (18·4) | 30 (15·7) |
| **2015–2019 (%)** | 4675 (38·8) | 3870 (37·8) | 487 (47·0) | 237 (40·1) | 81 (42·4) |
| **2020–2024 (%)** | 4866 (40·4) | 4146 (40·5) | 395 (38·1) | 245 (41·5) | 80 (41·9) |

SD, standard deviation. 2vHPV, bivalent HPV vaccine. 4vHPV, quadrivalent HPV vaccine. 9vHPV, nonavalent HPV vaccine. CIN2, cervical intraepithelial neoplasia grade 2. CIN3, cervical intraepithelial neoplasia grade 3

^a^Percentages may not total 100% because of rounding.

S-Table 2. Recurrence rates of cervical intraepithelial neoplasia grade 1 or worse (CIN1+), carcinoma, and indeterminate dysplasia by timing of HPV vaccination and cone margin positivity

| **HPV vaccination status** | **No. of recurrences** | **Person-years (py)** | **Incidence rate per 1000 py (95% CI)** | **Crude incidence rate ratio (95% CI)** | **Adjusted incidence rate ratio (95% CI)^a^** | **Vaccine effectiveness (95% CI)** |
| --- | --- | --- | --- | --- | --- | --- |
| **Unvaccinated** | 655 | 43,312 | 15·12 (14·01–16·33) | Reference | Reference | Reference |
| **Vaccinated irrespective of timing** | 43 | 8631 | 4·98 (3·69–6·72) | 0·33 (0·24–0·45) | 0·35 (0·26–0·48) | 65 (52–74)* |
| **Vaccinated post-excision** | 19 | 5007 | 3·79 (2·42–5·95) | 0·25 (0·15–0·39) | 0·27 (0·17–0·43) | 73 (57–83)* |
| **Vaccinated pre-excision** | 18 | 2788 | 6·46 (4·07–10·25) | 0·43 (0·25–0·68) | 0·46 (0·29–0·73) | 54 (27–71)* |
| **Women with positive cone margin** |  |  |  |  |  |  |
| **Unvaccinated** | 325 | 6045 | 53·76 (48·22–59·94) | Reference | Reference | Reference |
| **Vaccinated irrespective of timing** | 19 | 1259 | 15·09 (9·62–23·66) | 0·28 (0·17–0·45) | 0·33 (0·21–0·53) | 67 (47–79)* |
| **Vaccinated post-excision** | 8 | 660 | 12·12 (6·06–24·23) | 0·23 (0·1–0·45) | 0·25 (0·13–0·51) | 75 (49–87)* |
| **Vaccinated pre-excision** | 9 | 468 | 19·21 (10–36·93) | 0·36 (0·16–0·69) | 0·45 (0·23–0·88) | 55 (12–77) |
| **Women with negative cone margin** |  |  |  |  |  |  |
| **Unvaccinated** | 255 | 31,610 | 8·07 (7·14–9·12) | Reference | Reference | Reference |
| **Vaccinated irrespective of timing** | 20 | 6591 | 3·03 (1·96–4·7) | 0·38 (0·23–0·59) | 0·39 (0·25–0·62) | 61 (38–75)* |
| **Vaccinated post-excision** | 9 | 3901 | 2·31 (1·2–4·43) | 0·29 (0·13–0·55) | 0·29 (0·15–0·57) | 71 (43–85)* |
| **Vaccinated pre-excision** | 8 | 2099 | 3·81 (1·91–7·62) | 0·47 (0·2–0·95) | 0·52 (0·26–1·06) | 48 (-6–74) |

* Result with a p<0·05 at statistical power >80%.

^a^ Poisson regression models adjusted for age groups, cone margin positivity (full analysis set), and HPV detection, with stratification by year and gynaecology clinic of conisation.

S-Table 3. Recurrence rates of cervical intraepithelial neoplasia grade 2 or worse (CIN2+) and carcinoma by age at vaccination before excision

| **HPV vaccination status** | **No. of recurrences** | **Person-years (py)** | **Incidence rate per 1000 py (95% CI)** | **Crude incidence rate ratio (95% CI)** | **Adjusted incidence rate ratio (95% CI)^a^** | **Vaccine effectiveness (95% CI)** |
| --- | --- | --- | --- | --- | --- | --- |
| **Unvaccinated** | 513 | 35,105 | 14·61 (13·4–15·93) | Reference | Reference | Reference |
| **Vaccinated up to 18 years** | 1 | 553 | 1·81 (0·25–12·84) | 0·12 (0–0·69) | 0·15 (0·02–1·05) | 85 (-5–98) |
| **Vaccinated over 18 years** | 13 | 1844 | 7·05 (4·09–12·14) | 0·48 (0·26–0·83) | 0·55 (0·31–0·95) | 45 (5–69) |

* Result with a p<0·05 at statistical power >80%.

^a^ Poisson regression models adjusted for age groups, cone margin positivity, and HPV presence, with stratification by years and clinical sites of conisation.

S-Figure 1. Forest plot of adjusted incidence rate ratios (aIRR) with 95% confidence intervals (CI) and corresponding incidence rates (IR) for study covariates. A – Recurrence of cervical intraepithelial neoplasia grade 2 or worse (CIN2+) and carcinoma; B – Recurrence of any dysplasia and carcinoma.
